# An Atypical Case of Yersinia enterocolitica Infection in a Patient Suspected With Ulcerative Colitis Flare-Up

**DOI:** 10.7759/cureus.53780

**Published:** 2024-02-07

**Authors:** Priyam Doshi, Ghomathy Sivaram, Corey Sievers

**Affiliations:** 1 Internal Medicine, Western Reserve Hospital, Cuyahoga Falls, USA; 2 Gastroenterology, Western Reserve Hospital, Cuyahoga Falls, USA

**Keywords:** infectious colitis, gut microbiome, yersinia enterocolitica, inflammatory bowel syndrome, ulcerative colitis (uc)

## Abstract

Ulcerative colitis (UC), one of the two major inflammatory bowel diseases (IBD), is a chronic immune-mediated inflammatory disorder with varying degrees of colonic mucosal involvement. Patients often present with inflammation limited to the rectum, also known as ulcerative proctitis, proximal colonic involvement, or pancolitis which affects the entire colon. Clinical manifestations of UC flare-ups include hematochezia, diarrhea, and abdominal pain. *Yersinia enterocolitica*, an acute cause of infectious diarrhea, is usually caused by the ingestion of food products contaminated with toxins and pathogens. The most common clinical presentation of a patient with acute *Y. enterocolitica* infection is self-limiting gastroenteritis. Microbial properties such as tissue invasion and immunological capability may be associated with the development of chronic conditions such as UC. IBD has been extensively studied, but the inter-relationship between IBD and infectious causes of diarrhea is still up for debate. We present a case of atypical *Y. enterocolitica *infection with a long-standing history of UC that was initially misdiagnosed as an acute UC flare-up.

## Introduction

Inflammatory bowel disease (IBD), a chronic immune-mediated condition, is characterized by inflammation of the gastrointestinal system [1,2]. IBD commonly presents as ulcerative colitis (UC) and Crohn’s disease (CD). CD commonly affects any portion of the gastrointestinal tract, usually the terminal ileum and colon. In contrast to CD, the inflammation of UC is limited to that of the colonic mucosa [2].

UC is a relapsing and remitting chronic inflammatory disorder typically spanning from the rectum and continuously extending through the proximal colon [1,2]. Patients often present with inflammation limited to the rectum, also known as ulcerative proctitis, proximal colonic involvement, or pancolitis which affects the entire colon [1,2]. The human gastrointestinal mucosa comes in contact with millions of antigens through exposure from food, microbiome, or environmental sources [1,3]. Defect in the mucosal barrier increases luminal antigens’ permeability, causing gut hyperstimulation [1,4-8]. UC flare-up often presents with abdominal pain, diarrhea, and hematochezia. It peaks during early and middle adulthood, affecting men and women equally [2]. It is hypothesized that UC could be related to exposure to environmental and microbial risk factors leading to exaggerated immune responses and microbiota alterations in susceptible cases [4-6,9]. *Yersinia enterocolitica*, *Shigella dysenteriae*, *Salmonella *species, and *Campylobacter jejuni* are some of the commonly implicated infectious agents of UC [6].

*Y. enterocolitica* was first described in humans in 1939 by Schleifstein and Coleman in the United States [10]. It is a gram-negative, rod-shaped, non-spore-forming, facultative anaerobe found in water, food, and animals, with pigs as the main reservoirs [10,11]. A primary pathogenic event is caused by the colonization of *Yersinia *strains at the terminal ileum via M cells overlying Peyer’s patches, and the proximal colon [1,10-12]. The bacteria are transported across the epithelial barrier, picked up by phagocytes, and transported to the mesenteric lymph nodes, causing a wide range of gastrointestinal diseases including self-limiting gastroenteritis, pseudo-appendicitis, terminal ileitis, and mesenteric lymphadenitis [1,9,10,12].

This case report was previously presented as a meeting abstract at the 2023 American College of Gastroenterology on 23rd October 2023 (https://journals.lww.com/ajg/fulltext/2023/10001/s3221_an_atypical_case_of_yersinia_enterocolitica.1800.aspx).

## Case presentation

A 77-year-old woman with a pertinent history of UC since 1972 presented with four to five episodes of watery diarrhea, diffuse abdominal pain, and nausea for three days. The reason for considering the UC flare-up was the presence of blood-streaked stools before admission. She denied localized abdominal pain, fever, chills, or recent infection. The initial physical examination showed mild generalized abdominal tenderness without guarding or rigidity. Vitals were stable on presentation. Laboratory test results are presented in Table 1. C-reactive protein (CRP) and erythrocyte sedimentation rate (ESR) were within the normal range; they are helpful markers but not specific enough to rule out IBD [2]. Stool markers were not done.

**Table 1:** Pertinent laboratory values on admission g/dL = gram per deciliter, % = percentage, MCV = mean corpuscular volume, ESR = erythrocyte sedimentation rate, CRP = C-reactive protein, FL = femtoliter, mcL = microliter, mm/h = millimeter per hour, mg/L = milligram per liter, mmol/L = millimoles per liter

Laboratory Test	Result	References
Hemoglobin, g/dL	12.6	12-16
Hematocrit, %	37.9	36-48
MCV, FL	88	80-100
Platelet, mcL	211	138-367
WBC, mcL	8.8	3.6-10.3
ESR, mm/h	15	0-20
CRP, mg/L	3.7	<=9.9
Sodium, mmol/L	141	136-145
Potassium, mmol/L	2.8	3.4-5.1

**Figure 1:**
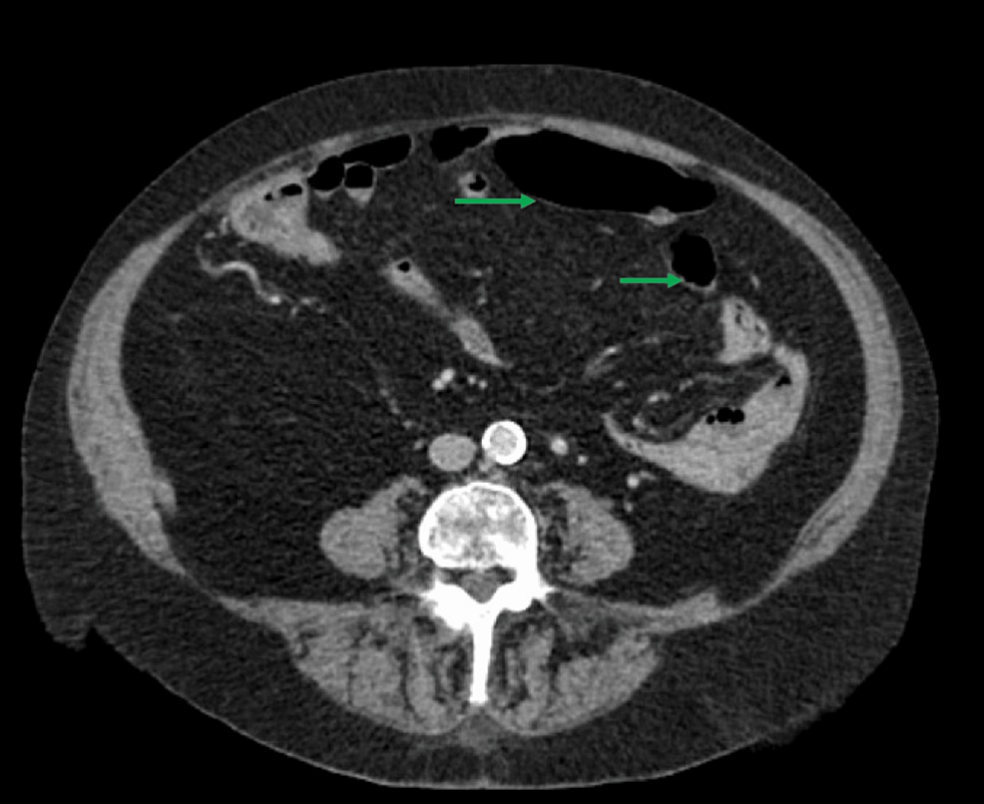
Axial section of CT scan of abdomen and pelvis during hospital admission The green arrows show a normal bowel wall thickness. The presence of mass or fluid collection was ruled out.

The last colonoscopy done in 2021 showed active UC pathology based on biopsy results (Figure 1). Images showed normal terminal ileum, erythema, and scarring in the right colon, left colon, rectum, and the presence of irregular margins with nodularity in the anal canal near the dentate line.

Considering clinical presentation and history of similar admissions, a case of early UC flare-up was suspected. The patient was started on intravenous (IV) Solu-Medrol® 40 milligrams (mg) twice daily and maintained IV fluids. A polymerase chain reaction (PCR) gastrointestinal panel was conducted to rule out an infectious cause of diarrhea. Results came back positive for *Y. enterocolitica*. By day 3, IV steroids were discontinued and the patient was transitioned to supportive care with IV fluids and nutritional support. By day 4 of hospitalization, diarrheal episodes improved, and diet was well tolerated. The patient was discharged on her home dose of Humira® 40 mg every two weeks and oral prednisone 5 mg once daily.

## Discussion

UC is a chronic immune-mediated inflammatory disorder with varying degrees of colonic mucosal involvement. Patients often present with inflammation limited to the rectum, also known as ulcerative proctitis, proximal colonic involvement, or pancolitis which affects the entire colon [2,13]. Symptoms tend to be gradual or sudden in onset [2]. Clinical manifestations of UC include hematochezia, diarrhea, and abdominal pain [1,2]. Common endoscopic findings include erythema, friable mucosa, pseudo-polyps, and continuous lesions. Fecal calprotectin (FC) levels are known to be related to the disease severity in UC patients [14]. Alternative non-invasive methods such as an FC assay could prevent aggressive testing such as colonoscopy and biopsy, possibly helping improve the patient’s quality of life [14]. Fecal lactoferrin (FL) is secreted by neutrophils in the inflamed intestine [15]. High FL levels were known to be associated with more chances of relapses and disease severity in patients with UC [15].

*Y. enterocolitica* infection is frequently associated with self-limiting gastroenteritis, but invasive characteristics are common in susceptible patients [6,10]. Contaminated food and water are known to be the major routes of *Y. enterocolitica* infection [1,3,10]. Most frequently isolated pathogenic strains belong to serogroups O:3, O:5,27, O:8, and O:9 [10].

The etiology of chronic inflammatory diseases like UC is multi-factorial with microbiota alterations secondary to microorganisms playing a key role [9]. A study out of Norway indicated an 11% rise in the incidence of long-term IBD in patients with preceding *Y. enterocolitica* infection [4-6]. This statistic strengthens the hypothesis that the incidence of IBD may involve an interaction between external infectious agents, host response, and genetic history [5].

Treatment modalities for UC depend on the severity of the condition. Initial therapy for most patients with acute severe UC includes systemic glucocorticoids [2,3]. Patients failing to improve within three to five days of IV glucocorticoids are known to benefit from medications such as infliximab, a tumor necrosis factor-alpha inhibitor (TNF-a), or cyclosporine, an immunosuppressive agent [1,2]. Colectomy is often the last resort [1,2]. *Y. enterocolitica* is treated mainly with supportive care including IV fluids, electrolyte, and nutritional replacement [16]. Most non-invasive presentations do not require antimicrobial therapy [16]. However, aggressive episodes have been shown to respond well to antibiotics such as aminoglycosides, trimethoprim-sulfamethoxazole, and cephalosporins [10,16].

## Conclusions

Our article discusses a case of initially suspected UC flare-up which was subsequently diagnosed as acute *Y. enterocolitica* infection. It has been observed in previous studies that *Y. enterocolitica* could lead to chronic GI symptoms secondary to tissue invasion and microbiota alterations. Hence, in patients with suspected UC flare-ups, clinicians must look for the incidence of concurrent causes of infectious diarrhea and consider other etiologies that could mimic IBD symptoms, such as in our case. Due to the increasing burden of IBD over the past decade, its association with infectious agents needs to be studied further.
